# Association between dizziness and future falls and fall-related injuries in older adults: a systematic review and meta-analysis

**DOI:** 10.1093/ageing/afae177

**Published:** 2024-09-19

**Authors:** Yuxiao Li, Rebecca M Smith, Susan L Whitney, Barry M Seemungal, Toby J Ellmers

**Affiliations:** Centre for Vestibular Neurology, Department of Brain Sciences, Imperial College London, Laboratory Block, Charing Cross Campus, London W6 8RF, UK; Centre for Vestibular Neurology, Department of Brain Sciences, Imperial College London, Laboratory Block, Charing Cross Campus, London W6 8RF, UK; Department of Physical Therapy, School of Health and Rehabilitation Science, University of Pittsburgh, 4200 Fifth Ave, Pittsburgh, PA 15260, USA; Centre for Vestibular Neurology, Department of Brain Sciences, Imperial College London, Laboratory Block, Charing Cross Campus, London W6 8RF, UK; Centre for Vestibular Neurology, Department of Brain Sciences, Imperial College London, Laboratory Block, Charing Cross Campus, London W6 8RF, UK

**Keywords:** vertigo, benign paroxysmal positional vertigo (BPPV), orthostatic hypotension, falls, prospective, systematic review, older people

## Abstract

**Background:**

Dizziness is common in older adults, especially in those attending falls services. Yet, the extent to which dizziness is associated with future falls has not been reviewed. This systematic review and meta-analysis assessed the association between dizziness and future falls and related injuries in older adults.

**Methods:**

EMBASE, CINAHL Plus, SCOPUS and PsycINFO databases were searched from inception to 5 February 2024. The review was registered on PROSPERO (registration ID: CRD42022371839). Meta-analyses were conducted for the associations of dizziness with future falls (including recurrent and injurious falls). Three meta-analyses were performed on different outcomes: any-type falls (≥1 falls), recurrent falls (≥2 falls) and injurious falls.

**Results:**

Twenty-nine articles were included in the systematic review (*N* = 103 306 participants). In a meta-analysis of 14 articles (*N* = 46 795 participants), dizziness was associated with significantly higher odds of any-type future falls (OR = 1.63, 95% CI = 1.44–1.84). In another meta-analysis involving seven articles (*N* = 5630 participants), individuals with dizziness also had significantly higher odds of future recurrent falls (OR = 1.98, 95% CI = 1.62–2.42). For both meta-analyses, significant overall associations were observed even when adjusted for important confounding variables. In contrast, a meta-analysis (three articles, *N* = 46 631 participants) revealed a lack of significant association between dizziness and future injurious falls (OR = 1.12, 95% CI = 0.87–1.45).

**Conclusions:**

Dizziness is an independent predictor of future falls in older adults. These findings emphasise the importance of recognising dizziness as a risk factor for falls and implementing appropriate interventions.

## Key Points

- This systematic review and meta-analysis examined the association between dizziness and future falls and/or injurious falls in older adults.
- Older adults with dizziness had a significantly higher odds of future falls.
- No significant association was observed between dizziness and future injurious falls.
- Our findings emphasise the importance of assessing dizziness in falls prevention services.

## Introduction

Approximately 30% of older adults fall at least once a year [1, 2]. Falls and related injuries lead to increased disability, hospitalisation, institutionalisation and mortality [1, 3, 4], resulting in poorer quality of life and greater healthcare costs [5]. A number of modifiable risk factors have been identified that can be managed to reduce the risk of falling [6].

The 2022 World Falls Guidelines highlighted dizziness as one such potentially modifiable risk factor [6]. Dizziness is a nonspecific term used to describe different feelings or sensations, such as vertigo, imbalance, light-headedness, and disorientation [7]. It is a common complaint in older adults: It affects over 50% of people older than 65 years and increases with age [8, 9]. Some of the commonest causes of dizziness in older adults are Benign Paroxysmal Positional Vertigo (BPPV) and cardiovascular issues, namely orthostatic hypotension (OH) [10]—both of which can be treated through targeted interventions [11, 12].

Older adults with dizziness have been reported to be at a higher risk of falls and fall-related injuries [13–15]. For instance, two recent systematic reviews and meta analyses noted (i) that more than one-in-two people who fell had vestibular dysfunction on examination [16] and (ii) those with BPPV on examination were more likely to have previously fallen [17]. However, these two reviews did not specifically focus on older adults (who are at the highest risk of falls). They also primarily explored retrospective falls rather than future falls (although Pauwels et al. [17] did show that BPPV treatment significantly reduced *future* falls). As falls resulting in head injury can cause combined peripheral and central vestibular dysfunction and dizziness [18, 19], this renders retrospective studies incapable of accurately determining the directionality of the association between dizziness and falls. Furthermore, this previous work did not explore the association between dizziness and injurious falls.

Therefore, there is a need for evidence that summarises prospective studies assessing if baseline dizziness predicts future falls and fall-related injuries. Such understanding will help clinicians identify individuals at high risk of falls (and/or injurious falls) and aid the implementation of targeted falls prevention strategies among those experiencing dizziness. It will also provide empirical support for the recommendations presented within the 2022 World Falls Guidelines [6], given that the dizziness recommendations were based on expert opinion alone. The aim of this systematic review and meta-analyses was to determine the association between dizziness and future falls and injurious falls in older adults.

## Methods

### Protocol and registration

This systematic review and meta-analysis was performed according to the Preferred Reporting Items for Systematic Reviews and Meta-Analyses (PRISMA) guidance [20]. The protocol was prospectively registered in PROSPERO (International Prospective Register of Systematic Reviews) (registration ID: CRD42022371839).

### Search strategy and information sources

The review question was: ‘Is baseline dizziness a predictor of future falls and/or injurious falls in older people, independent of other known risk factors?’ An inclusive MEDLINE search strategy was developed with an experienced research librarian, and adapted for EMBASE, CINAHL Plus, SCOPUS and PsycINFO. The strategy used medical subject headings (MeSH) terms and relevant keywords on dizziness, falls, older people, risk or prospective, aiming to cover all articles on this topic. All databases were searched for English and Chinese language publications from the date of inception to 5 February 2024 (initial search conducted on 3 February 2023; updated search conducted on 5 February 2024). The detailed search strategy is provided in Supplementary Appendix A.

### Eligibility criteria and study selection

We included articles with the following criteria: (i) study design exploring the association between baseline dizziness and future falls (including any-type falls (≥1 falls), recurrent falls (≥2 falls) and fall-related injuries); (ii) participants aged 60 years or older^1^; (iii) follow-up period of at least 6 months and (iv) published in English or Chinese. Intervention studies, case reports, reviews, qualitative studies, conference abstracts and letters to the editor were excluded. In addition, studies that recruited participants with specific diseases or conditions other than dizziness were also excluded from this review. Note, future falls could be assessed either prospectively (via falls diaries/calendars) or retrospectively, at the end of the follow-up period (via retrospective recall).

All the retrieved articles were managed using Covidence software [21]. After duplicate removal, two independent reviewers (YL and TJE) screened the titles, abstracts and full texts of all selected articles based on the eligibility criteria. In the case of disagreements, the third reviewer (RMS) checked the study against the eligibility criteria of the review, and a final decision was made following discussion between all three reviewers. The reference lists of the included articles were searched to identify any other eligible studies.

### Data extraction

A pilot extraction form was developed and pilot-tested on three articles (by Y.L.). This was then revised through discussions with two other reviewers (T.J.E. and R.M.S.). Subsequently, the lead reviewer (Y.L.) independently performed the data extraction, and another reviewer (T.J.E.) verified all extracted data. Any discrepancies were discussed between two reviewers to reach a consensus.

The following data were extracted from the included articles: general study characteristics (first author, year of publication and country), participant characteristics (sample size, settings, age and gender), methods (follow-up duration, measurement or definition of dizziness and fall-related outcomes and covariates), effect size (odds ratios, risk ratios, incident rate ratios and hazard ratios) and significance (95% confidence interval [CI] and *P*-value). In the case of missing or unclear information, the authors were contacted. If key information (effect estimates of the association between dizziness and falls) remained missing or vague after attempted contact, those studies were excluded from the review (*N* = 4). However, if other study information was unavailable or unclear (such as the proportion of females, specific measures of dizziness and specific *P*-values), it was considered as ‘not reported’ based on the available information.

### Risk of bias assessment

We assessed the quality of included studies using the Risk of Bias in Non-randomised Studies of Exposures (ROBINS-E) tool [22] and visualised the assessments using ‘robvis’ [23]. ROBINS-E includes seven domains of bias: confounding, measurement of the exposure, selection of participants, postexposure interventions, missing data, measurement of outcome and selection of the reported result. Each domain was either categorised as low risk of bias, some concerns, high risk of bias or very high risk of bias. The overall risk of bias for each study was then recorded as the highest risk of bias for any domain. The risk of bias assessment was performed by one reviewer (Y.L.) and independently checked by another reviewer (T.J.E.). Any disagreements were resolved through a consensus discussion with a third reviewer (R.M.S.).

### Statistical analysis

Meta-analyses were performed separately for the effect of self-reported dizziness on any-type falls, recurrent falls and injurious falls, with outcomes pooled using random effects models to account for heterogeneity between studies. The odds ratio (OR) with 95% confidence intervals (95% CI) was the commonest measure of association used across studies. Therefore, we only included studies that reported ORs and 95% CIs (or provided these upon request) in meta-analyses. We included one study [24] that initially reported their effect size with relative ratios; however, we obtained the OR values after contacting the first author. As we wanted to answer if dizziness independently predicted future falls, we aimed to pool adjusted OR when possible. If a study presented more than one adjusted OR, we selected the maximally adjusted model for pooling. We included unadjusted OR for articles without adjusted confounding. Subgroup analyses were subsequently performed based on whether the studies included adjusted confounding variables or not.

We assessed heterogeneity using the Cochrane’s *Q* test and *I*^2^ statistic, and reported tau^2^ as an estimator of between-study variance. Egger’s asymmetry test and visual inspection of a funnel plot were conducted to evaluate publication bias for meta-analyses when at least 10 studies were included in that particular meta-analysis [25]. All analyses were performed using R (version 4.3.1) and the ‘metafor’ package (version 4.2.0).

We conducted sensitivity analyses in which each meta-analysis was repeated excluding extreme outliers [26]. We then conducted further subgroup analyses in which each meta-analysis was repeated separating (i) studies that reported falls outcomes retrospectively at the end of follow-up from those that assessed falls throughout the follow-up period (e.g. via falls diaries), and (ii) studies via their risk of bias [27]. Note, due to the injurious falls meta-analysis only containing three studies, these additional analyses were not conducted for this outcome due to insufficient datasets.

## Results

**Figure 1:**
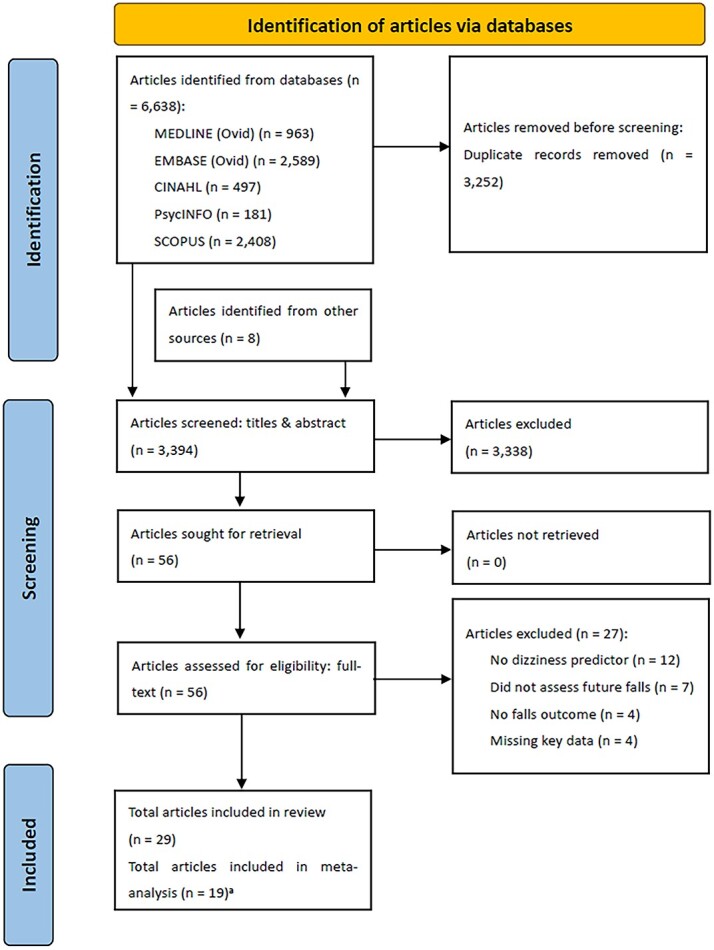
Study selection process. a These 19 articles generated 35 separate datasets to analyse in the meta-analysis, due to different outcomes used (any-type falls, recurrent falls, and fall-related injuries), and due to one article separately analysing data for 12 different European countries.

Figure 1 shows the PRISMA flow diagram of the study selection process. After removal of duplicates, 3394 articles were identified for title and abstract screening. Review of the titles and abstracts yielded 56 relevant articles for full-text screening. Twenty-nine articles met the inclusion criteria and were included in the systematic review (which included eight articles identified by searching the references of relevant papers).

Table 1 shows the study characteristics of the included studies. A total of 103 306 individuals (58% females) were studied across the included articles, with sample sizes ranging from 55 [28] to 32 619 [29] participants. Individual mean ages ranged from 62.9 to 83 years. Study populations included community-dwelling individuals, retirement community/village residents and community-dwelling outpatient clinics. Most of the studies were conducted in Europe or North America, followed by Australia, Asia and Africa.

**Table 1:** Study characteristics.

First author, year	Country/Region	Sample Size	Settings	Age Mean ± SD^a^	Gender (female)	Follow-up duration	Fall outcomes^b^	Effect size^c^ (95% CI), *P*-value
Chan, 2023 [29]	UK	32 619	Community-dwelling	69 ± 3	49.5%	9 years^d^	Occurrence of injurious falls (fall requiring inpatient medical care)	Unadjusted: **1.71 (1.12–2.60), *P* = 0.01** Adjusted model 1: **1.66 (1.09–2.53), *P* = 0.02** Adjusted model 2: 1.52 (0.99–2.33), *P* = 0.06
Chen, 2023 [30]	India	3257	Community-dwelling	68.5 ± 6.7	55.2%	3 years	Occurrence of injurious falls (fall resulting in pain, cuts, bruises, or fractures)	Unadjusted: **1.32 (1.08–1.62), *P* = 0.007** Adjusted model 1 and 2: 0.98 (0.78–1.23)
Claffey, 2022 [31]	Ireland	934	Community-dwelling	75	51.0%	≥2 years, average: 6.3 years (with follow-ups occuring every 2 years)	Occurrence of explained falls Occurrence of unexplained falls	AOH *vs* without OH predict unexplained falls: **2.01 (1.11–3.65), *P* = 0.022** AOH *vs* without OH predict explained falls: 0.93 (0.53–1.62), ***P* =** 0.797 SOH *vs* without OH predict unexplained falls**:** 0.86 (0.34–2.17), ***P* =** 0.754 SOH *vs* without OH predict explained falls**:** 0.90 (0.37–2.19), ***P* =** 0.812
Covinsky, 2001 [32]	USA	557	Retirement communities	81.6 ± 4.4	66.4%	1 year	Occurrence of any-type falls	Adjusted model 1: **1.96 (1.25–3.07)**^e^ Adjusted model 2: **1.83 (1.16–2.89)**^e^
Delbaere, 2010 [33]	Australia	500	Community-dwelling	77.9 ± 4.1	54.0%	1 year	Occurrence of any-type falls	**1.49 (1.01–2.18)** ^e^
Dinh, 2023 [34]	Germany, Netherlands	1015	Community-dwelling	77	50%	6 months	Occurrence of any-type falls	**2.3 (1.7–3.2)**, ***p* < 0.001**
Donnell, 2023 [35]	Ireland	2108	Community-dwelling	72.37	51.6%	≥2 years, average: 6.6 years for dizzy group at 7.0 years for non-dizzy (with follow-ups occuring every 2 years)	Occurrence of explained falls Occurrence of unexplained falls	Explained falls: Adjusted model 1: 1.22 (0.91–1.63), *P* = 0.189 Adjusted model 2: 1.14 (0.84–1.53), *P* = 0.408 Unexplained falls: Adjusted model 1: **2.04 (1.46–2.84), *p* < 0.001** Adjusted model 2: **1.82 (1.29–2.56), *P* = 0.001**
Faulkner, 2009 [36]	USA	8378	Community-dwelling	71 ± 3	100%	4 years	Fall rates (any-type falls)	*Relative Risk* Adjusted model 1: **1.29 (1.18, 1.41)**^e^ Adjusted model 2: **1.16 (1.06, 1.27)**^e^
Franse, 2017 [37]	12 European countries^f^	17 575	Community-dwelling	74.1 ± 6.8	55.8%	2 years	Occurrence of any-type falls	Switzerland: 1.84 (0.95–3.57) Denmark: **5.19 (2.76–9.77), *p* < 0.001** Sweden: 1.62 (0.85–3.08) Austria: 1.05 (0.66–1.69) Italy: **1.78 (1.10–2.87), *p* < 0.05** Netherlands: 1.16 (0.6–2.19) Germany: 1.21 (0.52–2.78) Belgium: 1.32 (0.87–1.99) Estonia: **1.45 (1.11–1.89), *p* < 0.01** France: 1.16 (0.77–1.77) Spain: 0.92 (0.61–1.39) Czech Republic: **1.97 (1.38–2.81), *p* < 0.001**
Gaßmann, 2009 [38]	Germany	622	Community-dwelling	Range 65–90	47.7%	6 months	Occurrence of any-type falls Occurrence of recurrent falls (≥2 falls in 6 months)	Any-type falls: **3.09 (1.90–5.03), *p* < 0.001** Recurrent falls: **4.51 (2.16–9.44), *p* < 0.001**
Gade, 2021 [39]	Denmark	241	Community-dwelling	Median (IQR) 82 (80; 86)	66.4%	1 year	Occurrence of any-type falls	**1.87 (1.10–3.19), *P* = 0.02**
Graafmans, 1996 [40]	Netherlands	354	Retirement communities	83 ± 6	85.0%	28 weeks	Occurrence of any-type falls Occurrence of recurrent falls (≥2 falls in 28 weeks)	Adjusted model 1: Any-type falls: **2.3 (1.3–3.8)**^e^ Recurrent falls: **2.3 (1.2–4.3)**^e^ Adjusted model 2: Any-type falls: **2.1 (1.2–3.7)**^e^ Recurrent falls: **2.1 (1.1–4.2)**^e^
Hansson, 2013 [28]	Sweden	55	Community physiotherapy centre	80 ± 11	74.5%	1 year	Occurrence of any-type falls	DHI total score: 2.28 (0.68–7.69) DHI functional: 1.22 (0.39–3.79) DHI emotional: 1.08 (0.35–3.38) DHI physical: 1.71 (0.54–5.41)
Heitterachi, 2002 [41]	Australia	70	Retirement villages	76.5 ± 5.9	80.0%	1 year	Occurrence of any-type falls	*Relative Risk* Dizzy on tilt: 0.82 (0.34–1.99) Dizzy when standing: 1.22 (0.78–1.92)
Himes, 2012 [42]	USA	10 755	Community-dwelling	75	63.6%	2 years	Occurrence of any-type falls Occurrence of injurious falls (fall requiring medical assistance)	Any-type falls: Adjusted model 1: **1.28 (1.14–1.45), *P* < 0.001** Adjusted model 2: **1.23 (1.09–1.39), *P* < 0.001** Injurious falls: Adjusted model 1: 1.03 (0.86–1.24) Adjusted model 2: 1.03 (0.85–1.24)
Kalula, 2016 [43]	South Africa	837	Community-dwelling	74.2 ± 6.4	76.5%	1 year	Occurrence of any-type falls Occurrence of recurrent falls (≥2 falls in 1 year)	Adjusted model 1: Any-type falls: **1.87 (1.19–2.93), *P* = 0.007** Adjusted model 2: Recurrent falls: **2.46 (1.14–5.33), *P* = 0.022**
Kwan, 2013 [44]	Taiwan, Hong Kong, and Australia	1456	Community-dwelling	76.3 ± 5.5	57.8%	1–2 years	Occurrence of any-type falls	*Incidence Rate Ratio* **1.43 (1.18–1.74), *P* = 0.007**
Luukinen, 1996 [45]	Finland	1016	Community-dwelling	76.1 ± 4.9	63.0%	2 years	Occurrence of recurrent falls (≥2 falls in 1 year)	**1.82 (1.09–3.05)** ^e^
Menant, 2013 [24]	Australia	516	Community-dwelling	79.7 ± 4.4	51.2%	1 year	Occurrence of recurrent falls (≥2 falls in 1 year)	*Odds Ratio* *^g^* Unadjusted: **1.73 (1.10–2.71)**^**e**^ Adjusted: 1.43 (0.90–2.28) *Relative Risk* Unadjusted: **1.55 (1.08–2.23)**^e^ Adjusted model 1: **1.54 (1.07–2.20)**^e^ Adjusted model 2: 1.33 (0.91–1.93)
O’Loughlin, 1993 [46]	Canada	409	Community-dwelling	Male: 73.7 Female: 75.5	62.8%	2 years	Number of any-type falls Number of injurious falls (falls resulting in ‘one or more injuries’)	*Incidence Rate Ratio* Unadjusted: Standing dizziness predict any-type falls: 1.6 Standing dizziness predict injurious falls: 0.8 Other dizziness predict any-type falls: 2.3 Other dizziness predict injurious falls: 1.6 Adjusted: Other dizziness predict any-type falls: **2.0 (1.3–2.8)**^e^
Pluijm, 2006 [47]	Netherlands	1365	Community-dwelling	75.3 ± 6.4	51.5%	3 years	Occurrence of recurrent falls (≥2 falls in 6 months)	Unadjusted: **2.05 (1.49–2.82)**^e^ Adjusted: **2.16 (1.47–3.17)**^e^
Sasidharan, 2020 [48]	India	1000	Community-dwelling	72.7 ± 7.2	56.8%	1 year	Occurrence of any-type falls	Unadjusted: **1.51 (1.10–2.06), *P* = 0.01**
Smith, 2022 [49]	UK	436	Rheumatology outpatient clinic	72.2 ± 7.3	68.6%	1 year	Occurrence of any-type falls	Unadjusted: **2.32 (1.43–3.80), *P* < 0.001** Adjusted: **2.46 (1.56–3.91), *P* < 0.001**
Tinetti, 1988 [50]	USA	336	Community-dwelling	78.3 ± 5.1	55.0%	1 year	Number of falls	*Relative Risk* 1.2 (0.8–1.6)
Tinetti, 2000 [51]	USA	1087	Community-dwelling	79.5 ± 5.2	73.0%	1 year	Occurrence of any-type falls	*Relative Hazard* Unadjusted: **1.35 (1.06–1.72)**^e^ Adjusted: 1.21 (0.94–1.55)
Tromp, 2001 [52]	Netherlands	1285	Community-dwelling	75.2 ± 6.5	51.1%	1 year	Occurrence of any-type falls Occurrence of recurrent falls (≥2 falls in 1 year)	Any-type falls: **1.5 (1.1–2.0)**^**e**^ Recurrent falls: **1.7 (1.1–2.6)**^**e**^
Valderrama-Hinds, 2018 [53]	Mexico	6247	Community-dwelling	69.9 ± 0.2	51.8%	11 years^h^	Occurrence of any-type falls	**1.41 (1.27–1.57)** ^e^
Welsh, 2019 [54]	UK	4386	Community-dwelling	62.9 ± 8.3	53.7%	6 years	Occurrence of any-type falls	Adjusted model 1: 3-year: 1.23 (0.96–1.58), *P* = 0.109 6-year: **1.69 (1.34–2.13), *P* < 0.001** Adjusted model 2: 3-year: 1.25 (0.97–1.60), *P* = 0.086 6-year: **1.70 (1.35–2.14), *P* < 0.001**
Woo, 2009 [55]	Hong Kong	3890	Community-dwelling	72	50.1%	2 years	Occurrence of recurrent falls (≥2 falls in 2 years)	Male: 1.34 (0.92–1.95) Female: **1.54 (1.17–2.04)**^e^

Abbreviation: OH = orthostatic hypotension; SOH = symptomatic orthostatic hypotension. AOH = asymptomatic orthostatic hypotension.

a Mean, unless otherwise stated. The standard deviation (SD) of age was not provided in six articles [31,34,35,42,46,55].

b Definitions of fall outcomes are summarised in Supplementary Appendix B.

c The effect sizes are reported as odds ratios (OR) by default, unless specified otherwise.

d Nine years between baseline and follow-up; but injurious falls were assessed each year.

e Indicates that specific *P*-values were not reported; however, the reported effect sizes are statistically significant.

f Twelve countries include Switzerland (*n* = 1345), Denmark (840), Sweden (946), Austria (1864), Italy (1364), Netherlands (1027), Germany (570), Belgium (1747), Estonia (2749), France (1771), Spain (1396) and Czech Republic (1956).

g The original data in this article were presented as relative ratios, we obtained the odds ratio values by contacting the authors.

h 11 years between baseline and follow-up; but falls were only assessed in final 2 years of follow-up. Bolded results indicate a statistically significant association.

### Assessment of dizziness

Several methods were used to assess dizziness (Supplementary Appendix B). The most commonly used method (26 articles) [24,29,30,31–34,35–40, 41–55] was self-report of dizziness, assessed with arbitrary questions such as ‘Have you experienced episodes of feeling dizzy, unsteady, or like you were spinning, moving, light-headed or faint?’, and a binary response of ‘yes’ or ‘no’. However, 16 of these articles assessed ‘dizziness’ without providing information on how dizziness was defined [29,30,33,34,37,38–40,42–45,48,50,52,55]. Additionally, the timeframe for recalling incidences of dizziness ranged from the very recent (i.e. any dizziness experienced during the previous 14 days) to longer-term experiences (i.e. any dizziness in the previous 2 years). Two studies specifically assessed dizziness experienced during episodes of orthostatic hypotension (OH, defined as a persisting drop in systolic blood pressure ≥ 20 mm Hg) [31,35]. Only one study [28] measured dizziness with the validated, multi-item Dizziness Handicap Inventory (DHI) scale [56].

### Assessment of falls and fall-related injuries

As shown in Table 1, a variety of falls-related variables (see Supplementary Appendix B for definitions used to define a ‘fall’) were assessed, including the occurence of any-type (≥1) falls [28, 32–34,36–44,46,48,49,51–54], falls rates (i.e. number of falls/person-years) [36], number of falls [47, 50], occasional falls (single fall) [38], recurrent falls (≥2 falls) [24,38,40,43,45,47,52,55], explained falls (accidental falls, due to slips or trips) or unexplained falls [31,35] and injurious falls [29,30,42,46]. Follow-up times ranged from 6 months [38] to 11 years [53], although for this latter study, falls were only assessed in the final two years of follow-up. Recurrent falls were defined as an individual falling at least twice within a designated period. However, the duration of this period varied from 6 months to 2 years across the studies.

Fifteen articles [28–32,34,35,37,38,41–43,53–55] recorded falls and related variables through retrospective recall at the end of the follow-up period, while the remaining fourteen articles [24,33,36,39,40,44–52] recorded falls and related variables through fall calendars or diaries (daily, weekly, monthly, or every 3 or 4 months).

### Main findings

#### Association between dizziness and any-type (≥1) future falls

The association between dizziness and any-type future falls was examined in 21 articles [28,32–34,36–44,46,48–54], and all but three studies [28,41,50] reported a significant association between dizziness and future falls. Among these, 11 studies [32,36,37,40,42–44,46,49,53,54] identified dizziness as an independent risk factor of falls after controlling for covariates (although different covariates were adjusted for different studies; see Supplementary Appendix C). In one study [51], dizziness was found to be associated with falls in the univariable analysis, but not in multivariable analysis after adjusting covariates. Moreover, six studies [33,34,38,39,48,52] reported univariable analyses only, and all reported that dizziness was associated with future falls.

**Figure 2:**
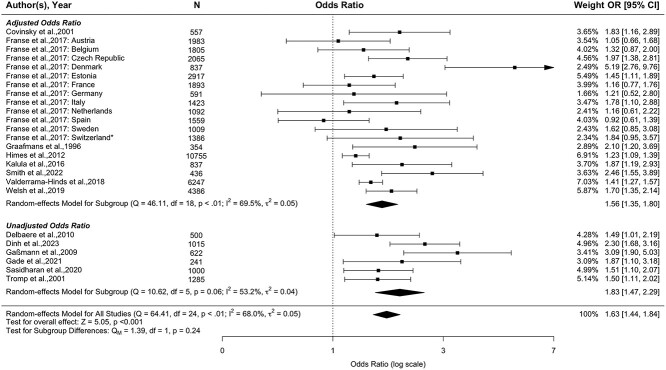
Forest plot of the association between dizziness and future any-type (≥1) falls (25 datasets from 14 studies). *Data analysed separately for 12 different European countries, as combined odd ratio values were not reported.

Figure 2 shows the forest plot of 14 articles included in the meta-analysis [32–34,37–40,42,43,48,49,52–54]. Twenty-five datasets were included within these 14 articles (46 795 participants), as one of these articles included 12 independent datasets from different European countries, and they did not report an overall effect size [37]. Older adults with dizziness had significantly higher odds of future falls (pooled OR, 95% CI = 1.63, 1.44–1.84, *P* < 0.001). Subgroup analyses showed that dizziness was significantly associated with future falls in unadjusted studies (6 datasets; pooled OR, 95% CI = 1.83, 1.47–2.29) and remained significant in adjusted studies (19 datasets; pooled OR, 95% CI = 1.56, 1.35–1.80). According to the funnel plot (see Supplementary Appendix D) and Egger test, there was no evidence for publication bias (*Z* = 1.60, *P* = 0.11). These results remained when removing an extreme outlier (pooled OR, 95% CI = 1.57, 1.41–1.75, *P* < 0.001; see Supplementary Appendix E, Figure S1). There was evidence of substantial heterogeneity (*I*^2^ = 68.0%, *P* < 0.01) that remained even after the removal of this outlier (*I*^2^ = 56.9%, *P* < 0.01; see Supplementary Appendix E, Figure S1).

#### Association between dizziness and future recurrent (≥2) falls

The association between dizziness and future recurrent falls was examined in 8 articles [24,38,40,43,45,47,52,55], and all studies found a significant relationship. Five studies [24,40,43,45,47] found that dizziness was independently associated with future recurrent falls, even after controlling for covariates.

**Figure 3:**
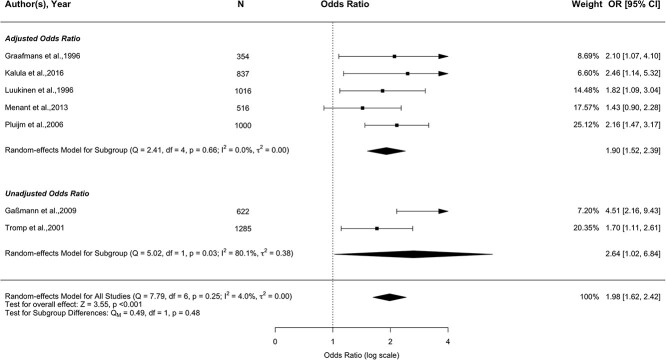
Forest plot of the association between dizziness and future recurrent (≥2) falls (seven studies).

Based on the meta-analysis of seven studies (Figure 3; 5630 participants) [24,38,40,43,45,47,52], dizziness was significantly associated with future recurrent falls (pooled OR, 95% CI = 1.98, 1.62–2.42, *P* < 0.001). There was no evidence of heterogeneity (*I*^2^ = 4.0%, *P* = 0.25). Subgroup analyses showed that dizziness was significantly associated with recurrent falls in unadjusted studies (2 datasets; pooled OR, 95% CI = 2.64, 1.02–6.84) and remained significant in adjusted studies (5 datasets; pooled OR, 95% CI = 1.90, 1.52–2.39). These results remained when removing an extreme outlier (pooled OR, 95% CI = 1.86, 1.52–2.27, *P* = 0.016; see Supplementary Appendix E, Figure S2).

#### Association between dizziness and future fall-related injuries

**Figure 4:**
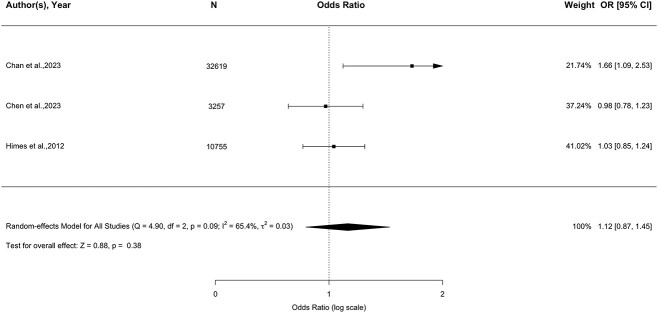
Forest plot of the association between dizziness and future fall-related injuries (three adjusted studies).

Four studies [29,30,42,46] examined the relationship between dizziness and future fall-related injuries. Two studies reported a significant association [29,30]; however, the other two studies indicated that the relationship was insignificant [42, 46]. Based on the meta-analysis of three adjusted studies (Figure 4; 46 631 participants) [29,30,42], dizziness was not associated with future fall-related injuries (pooled OR, 95% CI = 1.12, 0.87–1.45, *P* = 0.380). There was evidence of substantial heterogeneity (*I*^2^ = 65.4%, *P* = 0.09).

#### Association between dizziness due to orthostatic hypotension and future falls

Two studies [31,35] examined the association between dizziness due to orthostatic hypotension (OH) and future explained/unexplained falls. Donnell et al. [35] found that dizziness due to symptomatic OH (i.e. dizziness during standing due to OH) was independently associated with future unexplained falls (OR, 95% CI = 1.82, 1.29–2.56). However, symptomatic OH did not show a significant association with explained falls (OR, 95% CI = 1.14, 0.84–1.53). In contrast, Claffey et al. [31] reported that symptomatic OH was not associated with either explained (OR, 95% CI = 0.90, 0.37–2.19) or unexplained (OR, 95% CI = 0.81, 0.40–1.66) falls. However, asymptomatic OH was independently associated with unexplained falls (OR, 95% CI = 2.01, 1.11–3.65) but not explained falls (OR, 95% CI = 0.93, 0.53–1.62).

#### Quality of included studies

According to the ROBINS-E assessment of 29 studies (Supplementary Appendix F), three of the studies were assessed as having a low risk of bias [37,53,54], five studies had some concerns about bias [36,40,44,45,48], and the remaining studies (*n* = 21) were assessed as having a high risk of bias [24,28–35,38,39,41–43,46,47,49–52,55]. Overall, Domain 1 (bias due to confounding factors) and Domain 5 (bias due to missing data) were the main contributors to studies being rated as having a risk of bias. For Domain 1, we considered age and gender as the two most important confounding factors (given the associations between each of these variables and both dizziness and falls [57–59]). Articles were rated as having a low risk of bias if both age and gender have been controlled, ‘Weak No’ if only age or gender had been controlled, and ‘Strong No’ if neither age nor gender had been controlled. Thirteen studies [24,28,32–34,38,39,41,43,47,50,52,55] were evaluated to have high risk in Domain 1. Twelve studies [29–31,33,35,42,43,46,47,49,51,55] were assessed as having high risk of bias in Domain 5 due to lack of mention missing data (either rate of, or how the missing data were handled).

#### Additional subgroup analyses

When separating studies based on falls assessment—prospectively (e.g. falls diaries) *vs.* retrospectively reported at the end of follow-up—there was a lack of significant subgroup difference for any-type future falls (*P* = 0.58; see Supplementary Appendix E, Figure S3). There was, however, a significant subgroup difference for future recurrent falls (*P* = 0.03; see Supplementary Appendix E, Figure S4), with higher ORs for retrospective studies (although an overall significant positive association was observed for both subgroups). Further subgroup analyses separating studies based on risk of bias revealed no significant difference between risk of bias subgroups for either future any-type falls (*P* = 0.15; see Supplementary Appendix E, Figure S5) or future recurrent falls (*P* = 0.84; see Supplementary Appendix E, Figure S6); with a significant positive association observed for all subgroups.

## Discussion

This systematic review and meta-analysis evaluated the association between dizziness and both future falls and fall-related injuries in older adults. We found that baseline dizziness was associated with increased odds of future falls and recurrent falls. These associations remained even when controlling for important covariates, revealing that dizziness is an independent predictor of future falls in older adults. In contrast, associations with future fall-related injuries were not significant.

The present findings are in line with another recent systematic review and meta-analysis [17]. This previous review demonstrated that BPPV—one of the commonest causes of dizziness in older adults [60]—is associated with a 2.34-fold higher odds of having previously fallen (i.e. retrospective falls [17]). The present findings build on this work, revealing that dizziness more broadly is independently associated with future falls (with follow-up periods ranging from six months to 11 years). Examining the prospective association between baseline dizziness and future falls provides us with greater confidence about the directionality of this positive association.

The mechanisms underpinning the association between dizziness and future falls may be direct and/or indirect. For instance, dizziness due to peripheral vestibular dysfunction can result in greater postural instability, which may directly lead to a higher odds of falling [61–63]. Dizziness often also leads to fear of falling [64, 65], which can lead to maladaptive changes in behaviour and activity avoidance that may increase the risk of falling [66]. Future work should look to further scrutinise the exact mechanisms through which dizziness increases fall risks, to allow for the development of effective interventions.

Although we focused on self-reported dizziness symptoms, older adults with peripheral vestibular dysfunction may not perceive/experience dizziness [67] due to impaired vestibular perception. This is termed ‘vestibular agnosia’ (see [19, 68–71]). Vestibular agnosia is linked to impaired postural control and up to a 10-fold underdiagnosis of BPPV. Whilst the present findings highlight a clear relationship between perceived dizziness and future falls, falls are likely to be even more prevalent in individuals with vestibular agnosia as they will be unaware of, and therefore less able to compensate for, their risk of falling [19, 68]. Future work should therefore assess the relationship between self-reported dizziness and future falls, in combination with objective assessments of vestibular functioning including peripheral vestibular function (via standard clinical tests), vestibular perceptual function (via vestibular perceptual thresholds) and balance (via posturography).

Our results suggest that dizziness was not associated with future injurious falls. While the occurrence of falls may be directly influenced by impaired balance linked to dizziness/vestibular dysfunction, the occurrence of fall-related injuries may be additionally dependent upon extrinsic factors, such as environmental hazards (e.g. slippery or uneven walking surfaces) [72], or internal factors such as sarcopenia or physical frailty [73]. However, as the meta-analysis exploring the relationship between dizziness and future fall-related injuries only included three studies (all deemed to be at a high risk of bias), further examination is warranted.

The primary strength of this review was the use of future falls as the outcome, with follow-up periods of at least 6 months. This strengthens the reliability and directionality of the association between dizziness and falls. Prospective falls research, whereby the association between a baseline variable and the occurrence of future falls is assessed, is widely regarded as the most effective method to investigate factors that predict falls [74]. However, more than half of the included studies (15/29) assessed future falls via retrospective recall at the end of the follow-up, rather than through the gold-standard of falls diaries [75]. Despite this, subgroup analyses revealed comparable results between assessment modes when analysing any-type future falls (although the strength of association between dizziness and future recurrent falls was significantly stronger for retrospective assessment).

The findings presented should also be interpreted with regards to several further limitations. Most of the included studies were assessed as having a high risk of bias due to failure to adjust for age, as well as the lack of reporting of the missing data. However, the subgroup analyses revealed a lack of significant difference between studies when grouped based on risk of bias, which increases our confidence in the findings presented. Further, there was substantial heterogeneity across studies, particularly in the confounding variables controlled for and the assessment of dizziness. Finally, most included studies used arbitrary self-reported dizziness incidence or severity as the predictor variable (without clinical testing to confirm the cause of dizziness). It is therefore difficult to determine the exact mechanisms through which dizziness increases the risk of future falls. Future work should therefore examine how different causes of dizziness contribute to an individual's fall-risk (e.g. by comparing vestibular to cardiovascular causes).

Our results have important implications for clinical practice. The 2022 World Falls Guidelines recommended assessment of dizziness and vestibular disorders [6]. The findings from our review further underline the importance of including dizziness as part of a multicomponent falls assessment. We recommend that clinicians working in fall prevention should regularly ask older adults about any dizziness experienced. Those presenting with dizziness should undergo a thorough neuro-otological and cardiovascular assessment to identify the specific cause of symptoms. The results of the examination will assist the development of an effective treatment plan for modifiable risk factors for falls. For example, current evidence highlights that repositioning manoeuvres in older people with BPPV leads to a reduced risk of falling [17]. A recent paper describes the success and feasibility of adopting such targeted approach within a community falls service [76]. However, as common causes of dizziness can sometimes occur without conscious percept of symptoms, it is therefore also important to conduct objective assessments of neuro-otological and cardiovascular function in all older people with imbalance/deemed to be at risk of falling, not only those who report symptoms of dizziness [68].

## Conclusions

To our knowledge, this is the first systematic review and meta-analysis exploring the association between baseline dizziness and future falls and fall-related injury in older adults. Although there was no association between dizziness and injurious falls, dizziness was found to be an independent predictor of future (any-type and recurrent) falls in older adults. These findings emphasise the importance of investigating dizziness as a potential modifiable risk factor for falls and ascertaining whether interventions to reduce this risk are available (once the cause of dizziness has been identified). Notably, as dizzy older adults may present with multiple discrete causes of their symptoms, a multifactorial screening approach is likely needed to reduce falls in this group.

## Supplementary Material

Supplementary_file_afae177

## Footnotes

1 Inclusion criteria was listed as aged ≥65 in the original PROSPERO registration, but we chose to lower this to ≥60 due to a number of relevant papers using this lower inclusion age, and the World Falls Guidelines [6] also designing their recommendations for older adults aged ≥60.
