# Supplementary material for: Association between dizziness and future falls and fall-related injuries in older adults: a systematic review and meta-analysis
Source: Age Ageing. 2024 Sep 19;53(9):afae177. doi: 10.1093/ageing/afae177 (PMC11410394; doi:10.1093/ageing/afae177)
Supplement: Supplementary_file_afae177 [file supplementary_file_afae177.docx]

**Association between dizziness and future falls and fall-related injuries in older adults: a systematic review and meta-analysis**

**Appendix A.** Search strategy

**Appendix B.** Definitions and measures of dizziness and fall-related outcomes

**Appendix C.** Adjusted covariates in included studies

**Appendix D.** Funnel plot of Meta-analysis

**Appendix E.** Sensitivity and subgroup analyses

**Appendix F.** Risk of bias assessment

**Appendix G.** PRISMA guidelines checklist

**Appendix A. Search strategy**

| **Database** | **Search strategy** |
| --- | --- |
| **MEDLINE (Ovid)** | Ovid MEDLINE(R) and Epub Ahead of Print, In-Process, In-Data-Review & Other Non-Indexed Citations and Daily <1946 to February 03, 2023>  1 Dizziness/  2 exp Vertigo/  3 Vestibular Diseases/  4 (dizz* or light-headed* or lightheaded* or giddiness or giddy or vertigo* or (spinning adj2 sensation*) or bppv or vestibul*).mp.  5 1 or 2 or 3 or 4  6 exp Accidental Falls/  7 (fall or falls or fallen or falling or fell or slip* or trip* or stumbl* or stumble* or stumbling or tumbl* or tumble* or tumbling).mp.  8 6 or 7  9 exp Aged/  10 (old* or elder* or aged or ag?ing or geriatric* or pensioner* or senior*).mp.  11 9 or 10  12 risk/ or risk assessment/ or risk factors/  13 Prospective Studies/  14 (risk or predict or predicts or predictor or prediction* or cause or causes or prospective method* or prospective stud*).mp.  15 12 or 13 or 14  16 5 and 8 and 11 and 15 1054  17 limit 16 to English language 955  18 limit 16 to Chinese language 8 |
| **EMBASE (Ovid)** | Embase Classic+Embase <1947 to 2023 Week 05>  1 positional dizziness/ or dizziness/  2 exp vertigo/  3 vestibular disorder/  4 (dizz* or light-headed* or lightheaded* or giddiness or giddy or vertigo* or (spinning adj2 sensation*) or bppv or vestibul*).mp.  5 1 or 2 or 3 or 4  6 exp falling/  7 (fall or falls or fallen or falling or fell or slip* or trip* or stumbl* or stumble* or stumbling or tumbl* or tumble* or tumbling).mp.  8 6 or 7  9 exp aged/  10 (old* or elder* or aged or ag?ing or geriatric* or pensioner* or senior*).mp.  11 9 or 10  12 risk factor/  13 prospective study/  14 (risk or predict or predicts or predictor or prediction* or cause or causes or prospective method* or prospective stud*).mp.  15 12 or 13 or 14  16 5 and 8 and 11 and 15 2729  17 limit 16 to English language 2581  18 limit 16 to Chinese language 8 |
| **CINAHL** | S1 MH "Dizziness"  S2 (MH "Vertigo+")  S3 MH "Vestibular Diseases"  S4 dizz* or light-headed* or lightheaded* or giddiness or giddy or vertigo* or (spinning N2 sensation*) or bppv or vestibul*  S5 S1 OR S2 OR S3 OR S4  S6 MH "Accidental Falls"  S7 fall or falls or fallen or falling or fell or slip* or trip* or stumbl* or stumble* or stumbling or tumbl* or tumble* or tumbling  S8 S6 or S7  S9 MH "Aged+"  S10 old* or elder* or aged or ag#ing or geriatric* or pensioner* or senior*  S11 S9 or S10  S12 MH "Risk Factors"  S13 MH "Prospective Studies"  S14 risk or predict or predicts or predictor or prediction* or cause or causes or prospective method* or prospective stud*  S15 S12 or S13 or S14  S16 S5 and S8 and S11 and S15 513  S17 limit 16 to English language 496  S18 limit 16 to Chinese language 1 |
| **PsycINFO (Ovid)** | APA PsycInfo <1806 to January Week 4 2023>  1 exp Vertigo/  2 (dizz* or light-headed* or lightheaded* or giddiness or giddy or vertigo* or (spinning adj2 sensation*) or bppv or vestibul*).mp.  3 1 or 2  4 exp Falls/  5 (fall or falls or fallen or falling or fell or slip* or trip* or stumbl* or stumble* or stumbling or tumbl* or tumble* or tumbling).mp.  6 4 or 5  7 exp Older Adulthood/  8 (old* or elder* or aged or ag?ing or geriatric* or pensioner* or senior*).mp.  9 7 or 8  10 exp Risk Factors/  11 exp Prospective Studies/  12 (risk or predict or predicts or predictor or prediction* or cause or causes or prospective method* or prospective stud*).mp.  13 10 or 11 or 12  14 3 and 6 and 9 and 13 198  15 limit 14 to English language 181  16 limit 14 to Chinese language 0 |
| **SCOPUS** | ( TITLE-ABS-KEY ( dizz* OR light-headed* OR lightheaded* OR giddiness OR giddy OR vertigo* OR ( spinning W/2 sensation* ) OR bppv OR vestibul* ) ) AND ( TITLE-ABS-KEY ( fall OR falls OR fallen OR falling OR fell OR slip* OR trip* OR stumbl* OR stumble* OR stumbling OR tumbl* OR tumble* OR tumbling ) ) AND ( TITLE-ABS-KEY ( old* OR elder* OR aged OR aging OR ageing OR geriatric* OR pensioner* OR senior* ) ) AND ( ( TITLE-ABS-KEY ( risk OR predict OR predicts OR predictor OR prediction* OR cause OR causes ) OR TITLE-ABS-KEY ( "prospective method" OR "prospective methodology" OR "prospective study" ) ) ) AND ( LIMIT-TO ( LANGUAGE , "English" ) OR LIMIT-TO ( LANGUAGE , "Chinese" ) ) 2408 |

**Appendix B. Definitions and measures of dizziness and fall-related outcomes**

| **First author, year** | **Dizziness** | **Measures** | **Definitions** | **Fall outcomes** | **Measures** | **Definitions** |
| --- | --- | --- | --- | --- | --- | --- |
| Chan, 2023 | General dizziness | Incidence of dizziness from NHS medical records | No definition of dizziness provided. | Injurious falls | Medical records (assessed yearly) | Injurious falls were defined as falls requiring inpatient medical care. |
| Chen, 2023 | General dizziness | Yes/no question (NR) | No definition of dizziness provided. | Injurious falls | Yes/no question (reported at end of follow-up) | Participants were asked the following questions: “Think back over the last few years. Have you at any point in this time fallen down?” and “Did you have any injuries as a result of the fall?” |
| Claffey, 2022 | -Asymptomatic orthostatic hypotension (AOH)  -Symptomatic orthostatic hypotension (SOH) | Questions and orthostatic blood pressure test | Presence of dizziness, lightheadedness, or unsteadiness experienced during orthostatic blood pressure test | -Explained falls  -Unexplained falls | Yes/no question (previous 2 years; reported at end of follow-up) | Participants were asked “Have you had any falls since the last 2 years?” If they answered “yes” to this, participants were then asked, “Were any of these falls non-accidental, i.e. with no apparent or obvious reason?” This was used to inform the ‘Unexplained Falls’ variable. Explained falls were defined as accidental falls, due to slips or trips. |
| Covinsky, 2001 | Unbalanced or dizzy | Yes/no question (previous 1 year) | Participants were asked “Whether you had had trouble with either dizziness or balance during the past year”. | Falls | Yes/no question (reported at end of follow-up) | A fall was defined as unintentionally coming to rest on the ground. |
| Delbaere, 2010 | General dizziness | Yes/no question (NR) | No definition of dizziness provided. | Falls | Monthly falls diary | A fall was defined as “an unexpected event in which the person comes to rest on the ground, floor, or lower level.” |
| Dinh, 2023 | General dizziness | Yes/no question (NR) | No definition of dizziness provided. | Falls | Yes/no question (reported at end of follow-up) | No definition of falls provided. |
| Donnell, 2023 | Symptomatic orthostatic hypotension (OH)^c^ | Questions and orthostatic blood pressure test | Presence of unsteadiness experienced during orthostatic blood pressure test. | -Explained falls  -Unexplained falls | Yes/no question (previous 2 years; reported at end of follow-up) | Participants were asked “Have you had any falls since the last 2 years?” If they answered yes to this, participants were then asked, “Were any of these falls non-accidental, i.e., with no apparent or obvious reason?” This was used to inform the ‘Unexplained Falls’ variable. Explained falls were defined as accidental falls, because of slips or trips. |
| Faulkner, 2009 | Dizziness upon standing | Yes/no question (NR) | No definition of dizziness provided. | Fall rates | Fall diary (previous 4 months) | The definition of falls was “landing on the floor or ground, or falling and hitting an object like a table or a chair”. Incident fall rates were calculated by dividing the number of falls by woman-years (including recurring falls and corresponding person-years). |
| Franse, 2017 | General dizziness | Yes/no question (dizziness experienced in past 6 months) | Participants was asked “For the past 6 months at least, have you been bothered by dizziness, faints or blackouts?”. | Falls | Yes/no question (previous 6 months; reported at end of follow-up) | The rate of falling was defined as the presence of one or more self-reported falls in the 6 months. This was assessed by asking participants “For the past 6 months at least, have you been bothered by any of the health conditions on this card?”, of which falling was listed. |
| Gaßmann, 2009 | General dizziness | Yes/no question (NR) | No definition of dizziness provided. | -Falls  -Recurrent falls (≥2 falls in 6 months) | Yes/no question (reported at end of follow-up) | Participants were classified as ‘non-fallers’ with no reported falls during the 6 months before the follow-up-assessment, as ‘fallers’ with at  least one fall, and as ‘recurrent fallers’ with two or more falls. |
| Gade, 2021 | General dizziness | Yes/no question (NR) | No definition of dizziness provided. | Falls | Daily fall calendar | A fall was defined as “an unexpected event in which the participants come to rest on the ground floor or lower level”. |
| Graafmans, 1996 | Dizziness upon standing | Yes/no question (experienced in previous 6 months) | No definition of dizziness provided. | -Falls  -Recurrent falls (≥2 falls in 28 weeks) | Weekly fall diary | A fall was defined as “unintentionally coming to rest at a lower level or on the ground.” Two outcome measures were delineated: any falls (≥1 fall) and recurrent falls (≥2 falls). |
| Hansson, 2013 | Self-perceived handicap because of dizziness | The Dizziness Handicap Inventory (DHI) | Dizziness was measured by the Dizziness Handicap Inventory (DHI). | Falls | Yes/no question (contacted every 3 months) | A fall was defined as “an unexpected event in which the participants come to rest on the ground, floor, or lower level.” |
| Heitterachi, 2002 | -Dizzy on tilt  -Dizzy when standing | Yes/no question (experienced in previous 6 months) | -Participants were asked “if you felt dizzy or light headed during and after the tilt”.  -Participants were asked “whether you experienced dizziness or light-headedness when you stood up”. | Falls | Yes/no question (contacted every month) | A fall was defined as an event in which they “unintentionally came to rest on the ground or at a lower level.” |
| Himes, 2012 | General dizziness | Yes/no question (NR) | No definition of dizziness provided. | -Falls  -Fall-related injuries | Yes/no question (reported at end of follow-up period) | Falls were assessed via the question: “Have you fallen down in the last 2 years?” If yes, participants were asked by the question “In that fall (or in any of these falls if more than one fall was reported) did you injure yourself seriously enough to need medical treatment?”. |
| Kalula, 2016 | General dizziness or vertigo | Yes/no question (NR) | No definition of dizziness provided. | -Falls  -Recurrent falls (≥2 falls in 1 year) | Yes/no question (reported at end of follow-up period) | A fall was defined as an episode in which a person “unintentionally comes to rest on the ground, floor or other lower level with or without injury.” The definition included falls which resulted from contributing factors, such as syncope, but excluded falls due to a violent blow, an epileptic seizure, or the sudden onset of paralysis such as in a stroke. Recurrent falls were defined as the occurrence of more than one fall in the last year. |
| Kwan, 2013 | General dizziness | Yes/no question (NR) | No definition of dizziness provided. | Falls | Monthly fall diary | Falls were defined as “unintentionally coming to the ground or other lower level and other than a consequence of sustaining a violent blow, loss of consciousness, sudden onset of paralysis as in stroke or epileptic seizure”. |
| Luukinen, 1996 | General dizziness | Yes/no question (NR) | No definition of dizziness provided. | Recurrent falls (≥2 falls in 1 year) | Daily fall diary | A fall was defined as an “unexpected event when the person fell to the ground from any level, including falls on stairs and falls onto a piece of furniture.” Recurrent falls were defined as at least two falls taking place within 365 days. |
| Menant, 2013 | General dizziness | Yes/no question (since age of 60 years) | Participants were asked “Since the age of 60 years, have you suffered from the following symptoms: (i) dizziness or vertigo; and (ii) light-headedness when standing up from a seat or bed?” Participants were categorized into the dizzy group based on positive answers to one or both of these questions; the remainder were categorized as non-dizzy. | Multiple falls (≥2 falls in 1 year) | Monthly falls diary | A fall was defined as “an unexpected event in which the person comes to rest on the ground, floor, or lower level.” Multiple fallers were defined as those who fell at least twice during the 12-month follow-up period. |
| O'Loughlin, 1993 | -Dizziness upon standing  -Other dizziness (other than upon standing) | Yes/no question (experienced in previous 14 days) | Participants were asked two separate questions about dizziness experienced in the 14 days preceding the interview: (i) “whether you had experienced dizziness upon standing up quickly” and (ii) “whether you had had any other dizziness, vertigo, or light-headedness”. | -Falls (number)  -Injurious falls (number) | Monthly fall calendar | A fall was defined as “an event which results in a person’s coming to rest inadvertently on the ground or other lower level”. Injurious falls included falls in which the subject reported sustaining one or more injuries that resulted from the fall. |
| Pluijm, 2006 | General dizziness | Yes/no question (NR) | Participants were asked “whether you were dizzy regularly”. | Recurrent falls (≥2 falls in 6 months) | Weekly fall calendar | A fall was defined as “an unintentional change in position resulting in coming to rest at a lower level or on the ground”. A ‘recurrent faller’ was defined as a subject who fell at least twice within a 6-month period. |
| Sasidharan, 2020 | General vertigo | Yes/no question (NR) | No definition of dizziness provided. | -Falls  -Recurrent falls (≥2 falls in 1 year) | Fall diary (frequency of completion not reported) | A fall was defined as “unintentionally coming to the ground or some lower level and other than as a consequence of sustaining a violent blow, loss of consciousness, sudden onset of paralysis as in stroke or an epileptic seizure”. A recurrent fall was defined as falling of two or more times during the follow-up period of 1 year. |
| Smith, 2022 | General dizziness | Yes/no question (NR) | Participants were asked “Whether you had had experienced dizziness”. | Falls | Monthly falls diary | Participants were asked to report any slip or trip in which they lost balance and landed on the floor, ground or lower level. A fall was defined as “an unexpected event in which the participants come to rest on the ground, floor, or lower level”. |
| Tinetti, 1988 | General dizziness | Yes/no question (experienced in previous 2 years) | No definition of dizziness provided. | Falls (number) | Fall diary (frequency of completion not reported) | A fall was defined as “unintentionally coming to rest on the ground or at some other lower level, not as a result of a major intrinsic event or overwhelming hazard (something that would result in a fall by most young, heathy persons). |
| Tinetti, 2000 | Chronic dizziness (presence for at least 1 month) | Yes/no question (experienced in previous 2 months) | The presence of chronic dizziness was ascertained using a two-step process. First, participants had to respond positively to the question, “During the past 2 months, have you had episodes of feeling dizzy, unsteady, or like you were spinning, moving, light-headed, or faint?” Second, for the dizziness to be considered chronic, the participant had to report its presence for at least 1 month. | Falls | Daily fall calendar | No definition of falls provided. |
| Tromp, 2001 | General dizziness | Yes/no question (NR) | No definition of dizziness provided. | -Falls  -Recurrent falls (≥2 falls in 1 year) | 3-Month fall calendar | A fall was defined as “an unintentional change in position resulting in coming to rest on the ground or other lower level”. Recurrent fallers were defined as those subjects with two or more falls during one year of follow-up. |
| Valderrama-Hinds, 2018 | Vertigo | Yes/no question (experienced in previous 2 years) | Participants were asked “During the last two years have you frequently had fainting spells or vertigo?”. | Falls | Yes/no question (previous 2 years; reported at end of follow-up period) | Falls were assessed by the question, “Have you fallen down in the last two years?”. A positive response was followed by, “Approximately how many times has this happened?” |
| Welsh, 2019 | General dizziness or unsteadiness | Yes/no question (experienced in previous 3 months) | Participants were asked “Whether you had had experienced dizziness or unsteadiness over the past three months”. | Falls | Yes/no question (previous 3 months; reported at both 3-year and 6-year follow-up) | Respondents were asked if they had “suffered from a fall or falls in the past three months”; yes or no responses were recorded. |
| Woo, 2009 | Dizziness | Yes/no question (NR) | No definition of dizziness provided. | Recurrent falls (≥2 falls in 2 years) | Yes/no question (contacted every 4 months) | A fall was defined as “any unexpected loss of balance resulting in coming to rest on the ground.” Recurrent fallers were defined as those subjects with two or more falls. |

**Abbreviation:** NR = not reported; OH = orthostatic hypotension; SOH = symptomatic orthostatic hypotension. AOH = asymptomatic orthostatic hypotension.

**Appendix C.** **Adjusted covariates in included studies**

| **First author, year** | **Adjusted covariates** |
| --- | --- |
| Chan, 2023 | Adjusted model 1: age and gender  Adjusted model 2: age, gender, whether the participant lives alone, dementia, depression, central nervous system lesion, Parkinson’s disease, osteoarthritis, handgrip strength, choice reaction time, presence of abnormal sleeping duration, and alternated sleep phase |
| Chen, 2023 | Adjusted model 1: age, gender, nutritional status, fall history at baseline, education, chronic conditions, fatigue, widespread pain, poor memory, sleep problems, and Timed up and Go Test  Adjusted model 2: age, gender, nutritional status, fall history at baseline, education, chronic conditions, fatigue, widespread pain, poor memory, sleep problems, Timed up and Go Test, nutritional status*female |
| Claffey, 2022 | Age, gender, heart disease, frailty status, cognitive impairment, chronic disease burden, and length of follow-up |
| Covinsky, 2001 | Adjusted model 1: abnormal mobility  Adjusted model 2: abnormal mobility, and occurrence of a fall in previous year |
| Delbaere, 2010 | No adjusted analysis performed |
| Dinh, 2023 | No adjusted analysis performed |
| Donnell, 2023 | Adjusted model 1: follow-up time  Adjusted model 2: age, gender, educational attainment, alcohol excess, heart disease, polypharmacy, and chronic disease burden |
| Faulkner, 2009 | Adjusted model 1: age, fall history, and recruitment site/clinic  Adjusted model 2: age, fall history, recruitment site/clinic, BMI, height, waist-to-hip circumference, stroke, Parkinson's Disease, diabetes, arthritis, self-rated health, fear of falling, visual acuity, depth perception, contrast sensitivity, Central nervous system active medications (benzodiazepines, antidepressants and antiepileptics), number of instrumental activities of daily living with difficulty, tandem stand balance with eyes open/closed, faster usual walking speed, chair-stand time, rapid stepping number completed in 10s, grip strength, physical activity, frequency goes outdoors, alcohol consumption, smoking status, hours per day spent on feet, and hours per week does household chores |
| Franse, 2017 | Age (≥80 years), gender, education level, living alone, self-rated health, mobility limitation, activities of daily living (ADL) limitation, impaired vision, depression, impaired cognition, and self-reported falling at baseline |
| Gaßmann, 2009 | No adjusted analysis performed |
| Gade, 2021 | No adjusted analysis performed |
| Graafmans, 1996 | Adjusted model 1: age and gender  Adjusted model 2: immobility  Adjusted model 3: history of stroke, poor mental state, and orthostatic hypotension |
| Hansson, 2013 | No adjusted analysis performed |
| Heitterachi, 2002 | No adjusted analysis performed |
| Himes, 2012 | Adjusted model 1: age, gender, race, obese category 1 (BMI 30.0–34.9 kg/m2), obese category 2 (BMI 35.0–39.9 kg/m2), and obese category 3 (BMI ≥ 40.0), underweight, body limitation, pain, and vision problem  Adjusted model 2: age, gender, race, obese category 1, obese category 2, and obese category 3, underweight, body limitation, pain, vision problem, diabetes mellitus, stoke, and arthritis |
| Kalula, 2016 | Adjusted model 1: previous falls, ethnicity, cognitive function  Adjusted model 2: previous falls, ethnicity, cognitive function, marital status, Timed Up and Go test |
| Kwan, 2013 | Age, gender |
| Luukinen, 1996 | Gender, urinary urgency, poor pulse rise 30s after standing up, frequent fear of falling, previous falls |
| Menant, 2013 | Adjusted model 1: medication  Adjusted model 2: neck and back pain, Goldberg anxiety scale, and physiological profile assessment |
| O'Loughlin, 1993 | Age, ≥2 different activities in past week, ≥10 activities in past week, daily alcohol consumption, days of limited activity, days spent in bed, trouble walking 400m, trouble bending down, and taking heart medicine |
| Pluijm, 2006 | ≥2 falls in the previous year, functional limitations, grip strength, body weight, fear of falling, dogs or cats in household, education ≥11 years, alcohol use, alcohol use × education, and ≥2 falls in previous fall × fear of falling |
| Sasidharan, 2020 | Age, gender, living arrangement, parkinsonism, arthritis, urinary symptoms, constipation, knee pain, paraesthesia of feet, history of fall in the previous year, dependence in basic and instrumental activities of daily living, use of assistive devices for movement, cognitive impairment, depression, use of antihypertensive medications and benzodiazepines |
| Smith, 2022 | Age, relationship status, Pain (visual analogue scale), and medicine for anxiety/depression |
| Tinetti, 1988 | No adjusted analysis performed |
| Tinetti, 2000 | Age, gender, depressive symptoms, self-rated health, falls self-efficacy, social activities, race, housing stratum, education, number of chronic conditions, number of medications, and any hospitalizations in previous year |
| Tromp, 2001 | No adjusted analysis performed |
| Valderrama-Hinds, 2018 | Age, gender, marital status, locality size (population), education years, BMI category, comorbid conditions, pain, urinary incontinence, vision problem, any lower extremity functional limitation, ADL disability, physical activity, depressive symptoms |
| Welsh, 2019 | Adjusted model 1: age, gender, number of pain sites, education, income adequate, occupational class, Index of Multiple Deprivation divided into quintiles, hearing deficit, visual deficit, Charlson Comorbidity Index score, BMI, depression, cognitive complaint, medication, analgesics, non-steroidal anti-inflammatory drug, physical functioning, and previous fall  Adjusted model 2: age, gender, widespread pain, education, income adequate, occupational class, Index of Multiple Deprivation, hearing deficit, visual deficit, Charlson Comorbidity Index score, BMI, depression, cognitive complaint, medication, analgesics, non-steroidal anti-inflammatory drug, physical functioning, and previous fall |
| Woo, 2009 | No adjusted analysis performed |

**Appendix D. Funnel plot of dizziness and risk of future any-type falls for evaluating publication bias.**

**
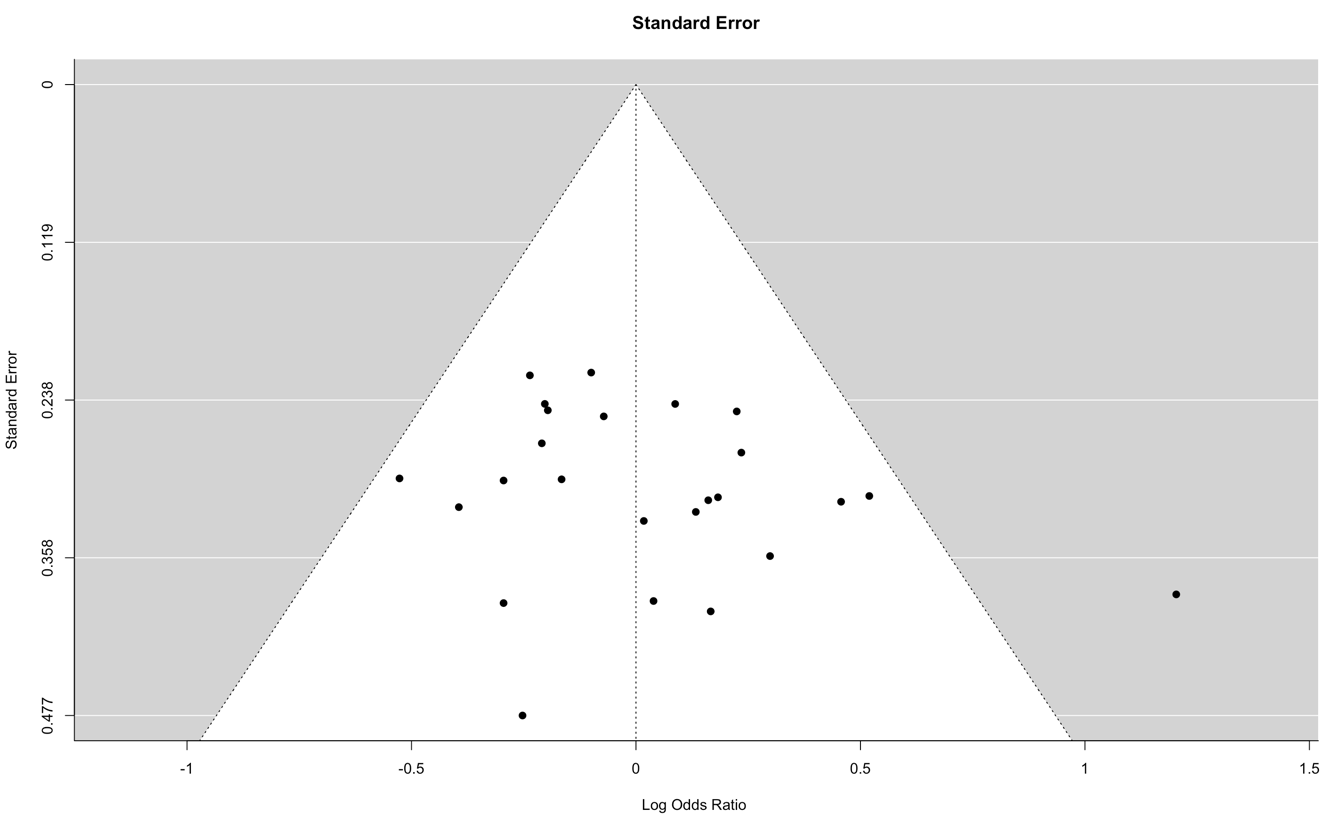
**

Both visual inspection for asymmetry and the results of Egger’s linear regression test (Z = 1.60, *P* = 0.11) suggest a lack of publication bias (25 datasets).

**Appendix E. Sensitivity and subgroup analyses**

**Figure 1. Forest plot of the association between dizziness and future any-type falls, excluding the outlier** (Franse et al., 2017: Denmark)


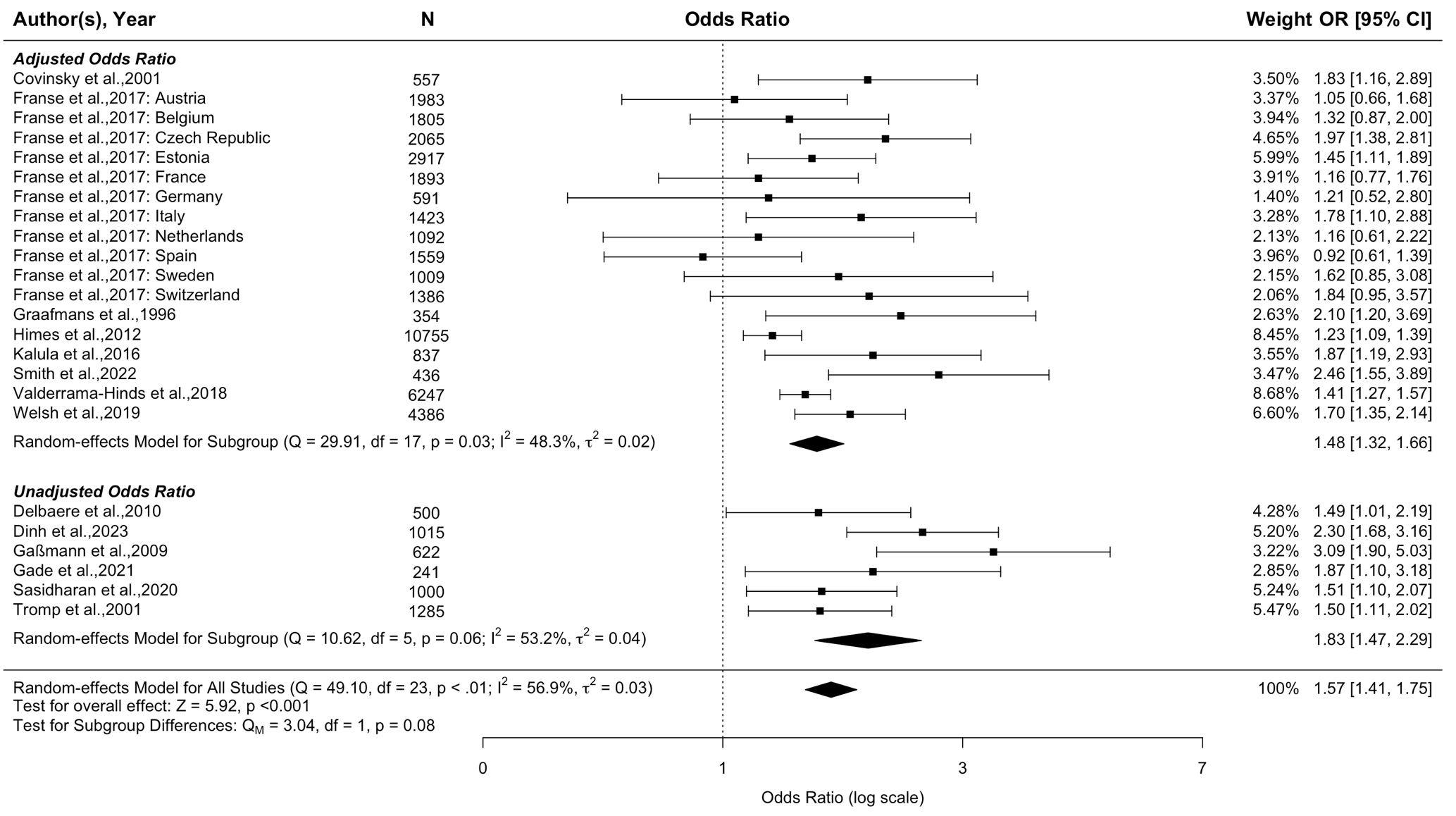


**Figure 2. Forest plot of the association between dizziness and future recurrent falls, excluding the outlier** (Gaßmann et al., 2009)


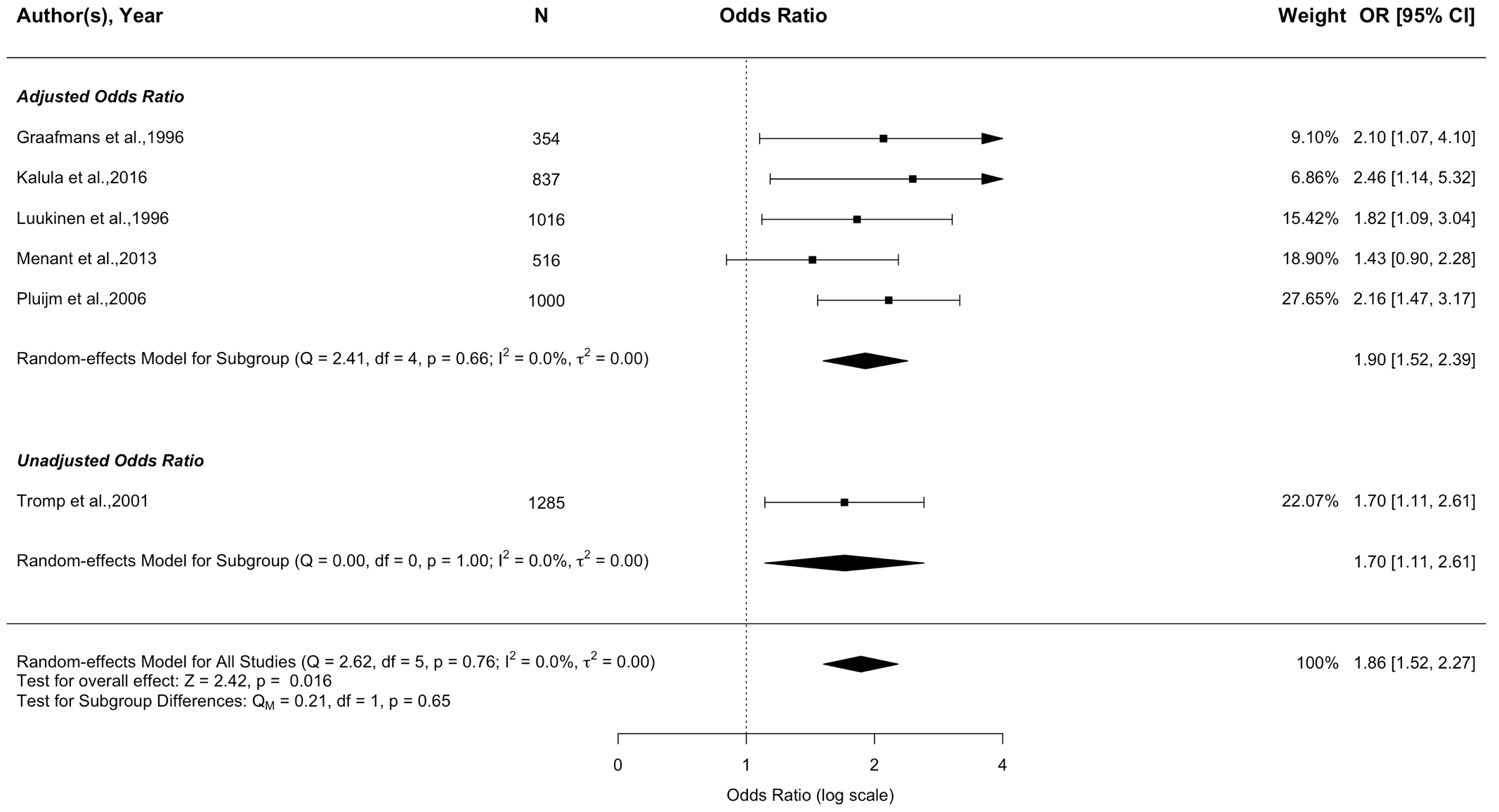


**Figure 3. Forest plot of the association between dizziness and future any-type falls, assessed prospectively (via falls diaries) *vs.* retrospectively (at the end of the follow-up)**


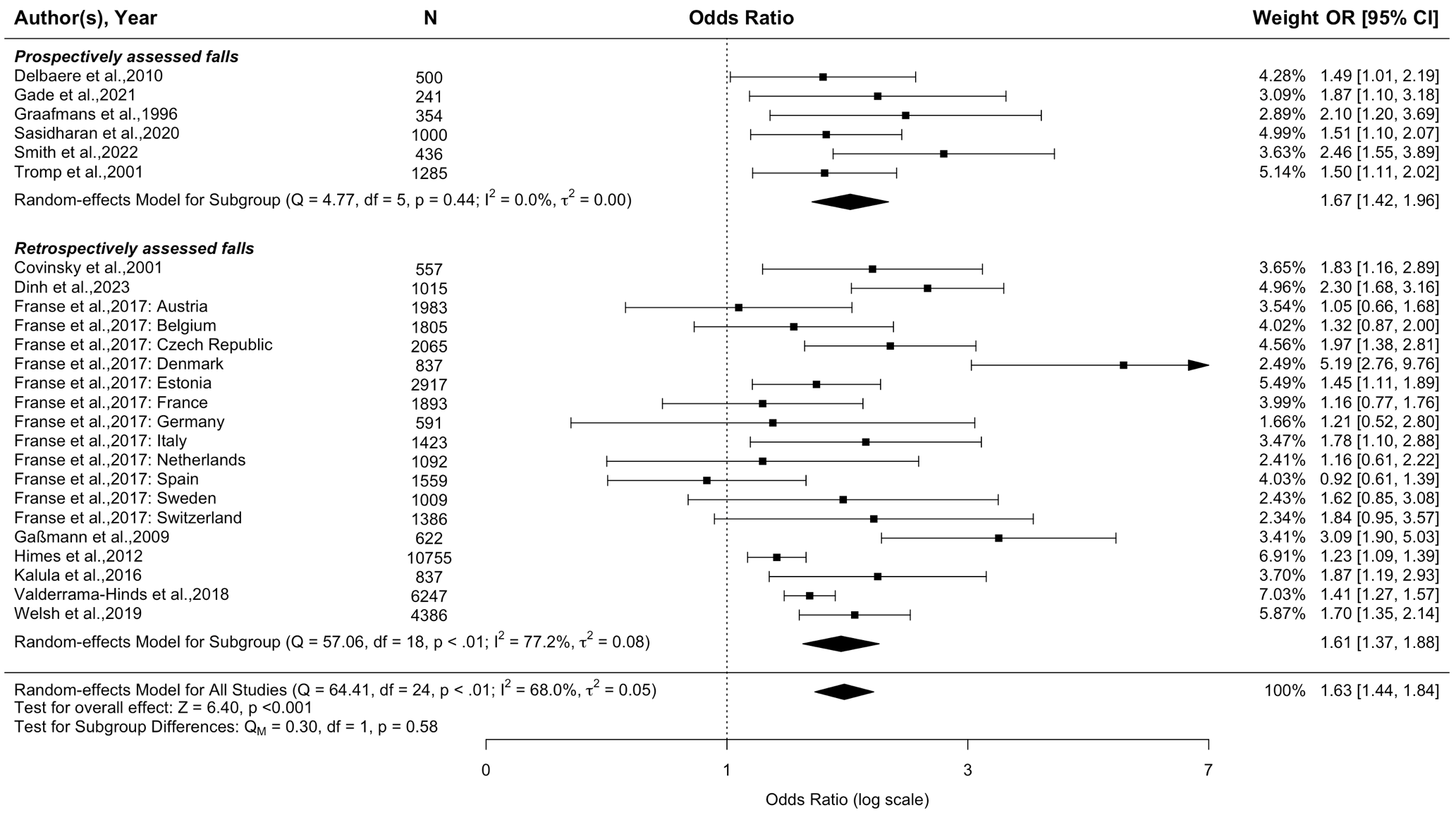


**Figure 4. Forest plot of the association between dizziness and future recurrent falls, assessed prospectively (via falls diaries) *vs.* retrospectively (at the end of the follow-up)**


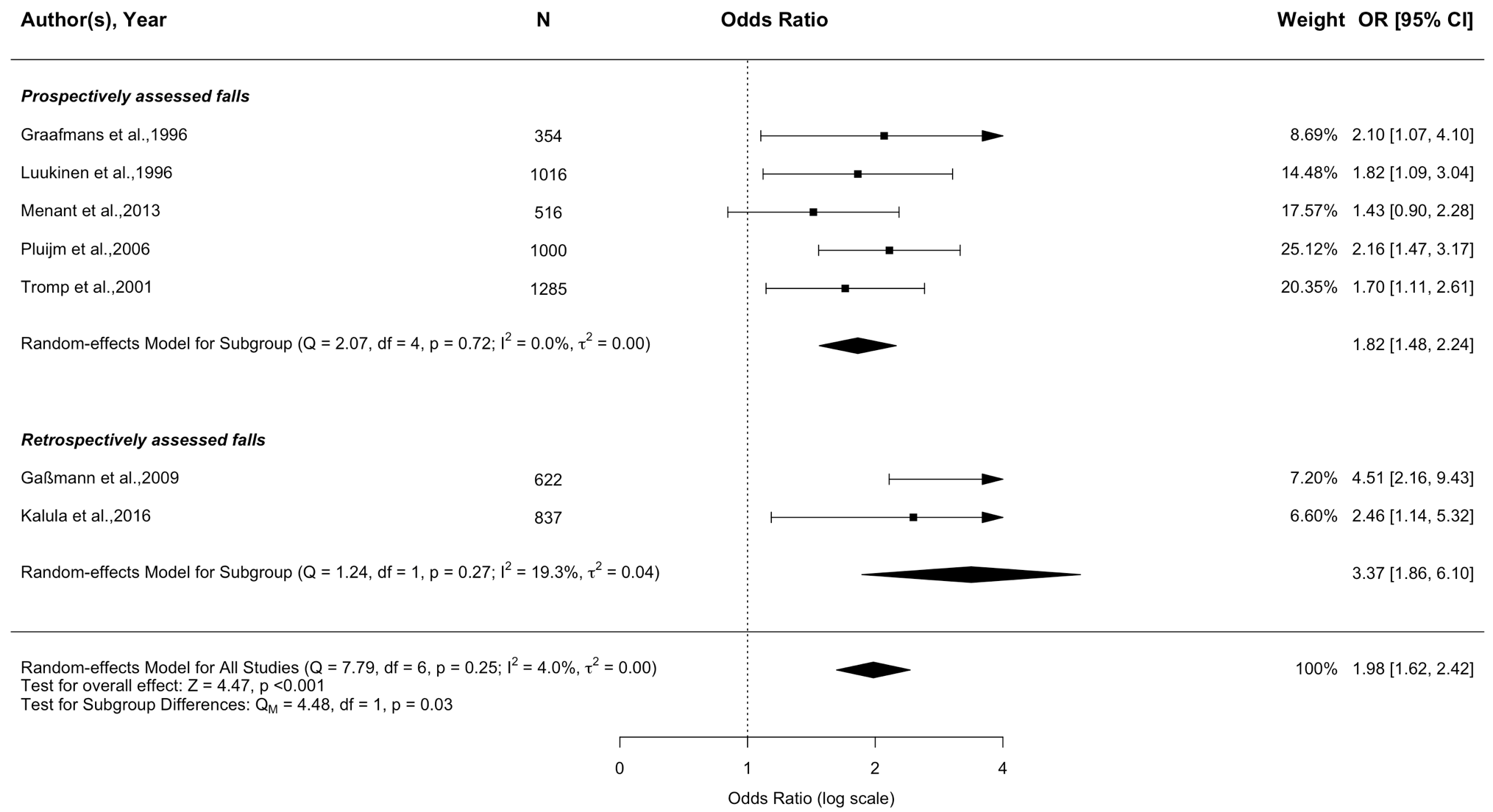


**Figure 5. Forest plot of the association between dizziness and future any-type falls, separated by risk of bias**


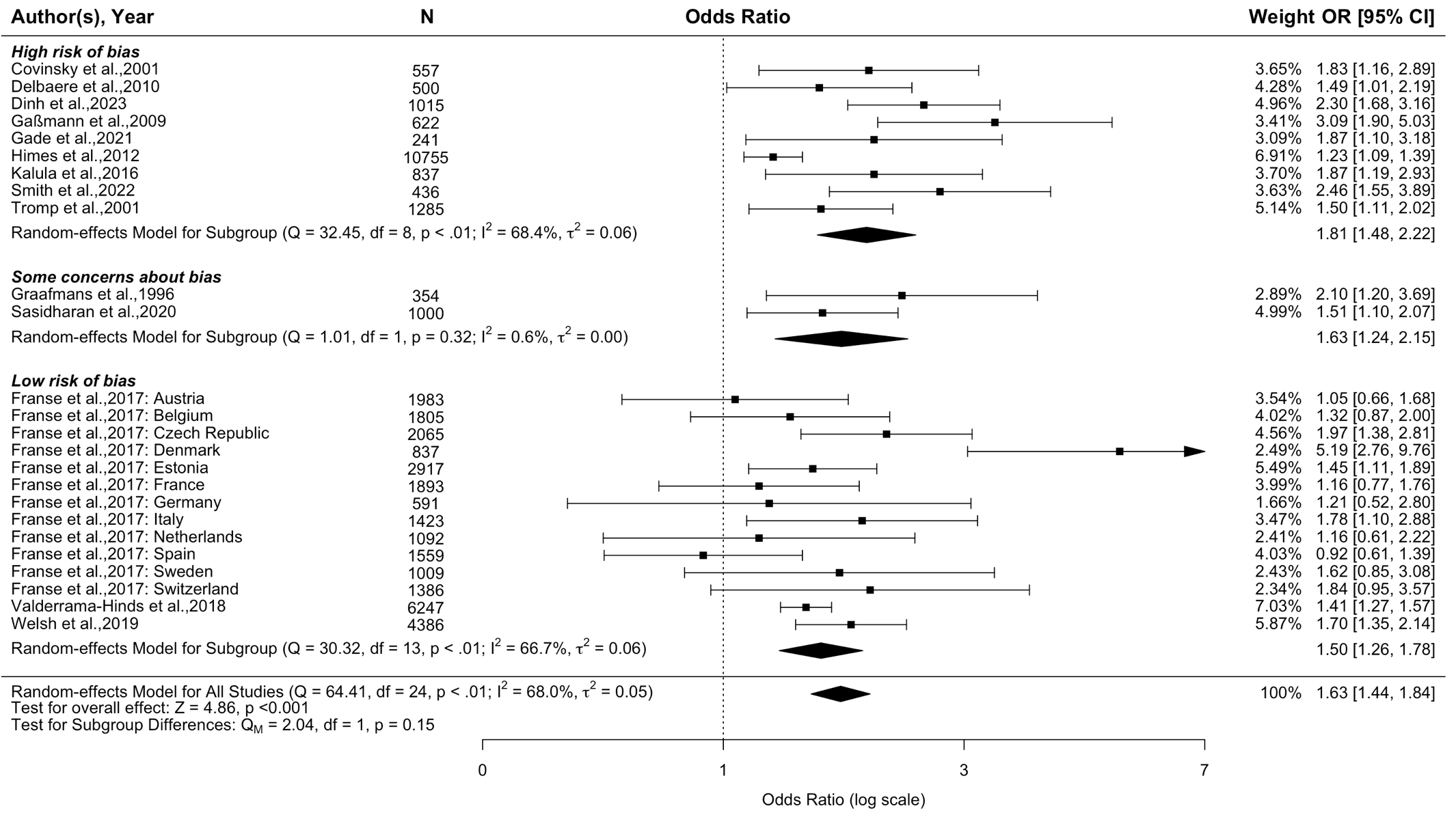


**Figure 6. Forest plot of the association between dizziness and future recurrent falls, separated by risk of bias**


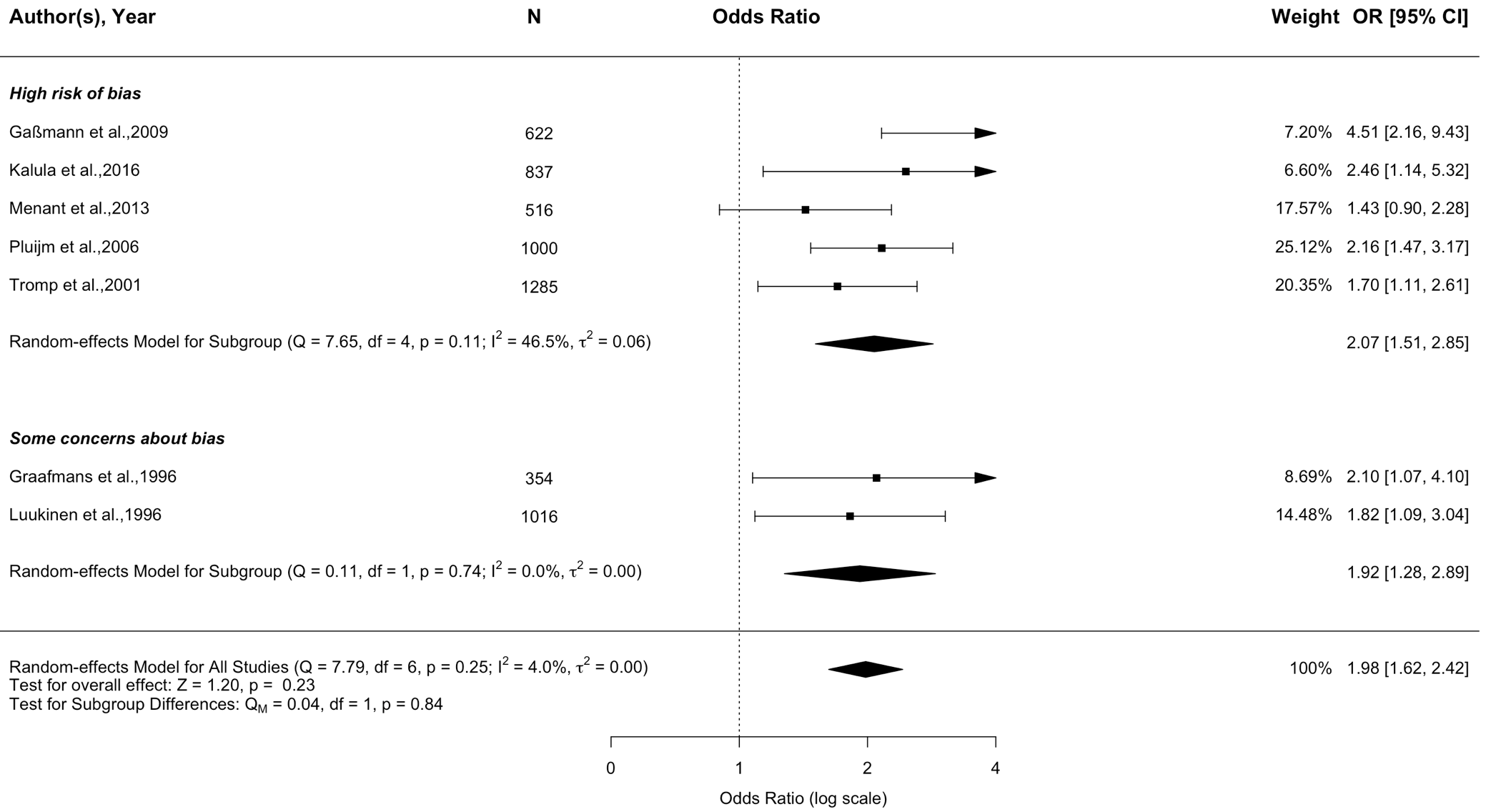


**Appendix F. Risk of bias assessment according to the Risk of Bias in Non-randomized Studies of Exposures (ROBINS-E) tool**


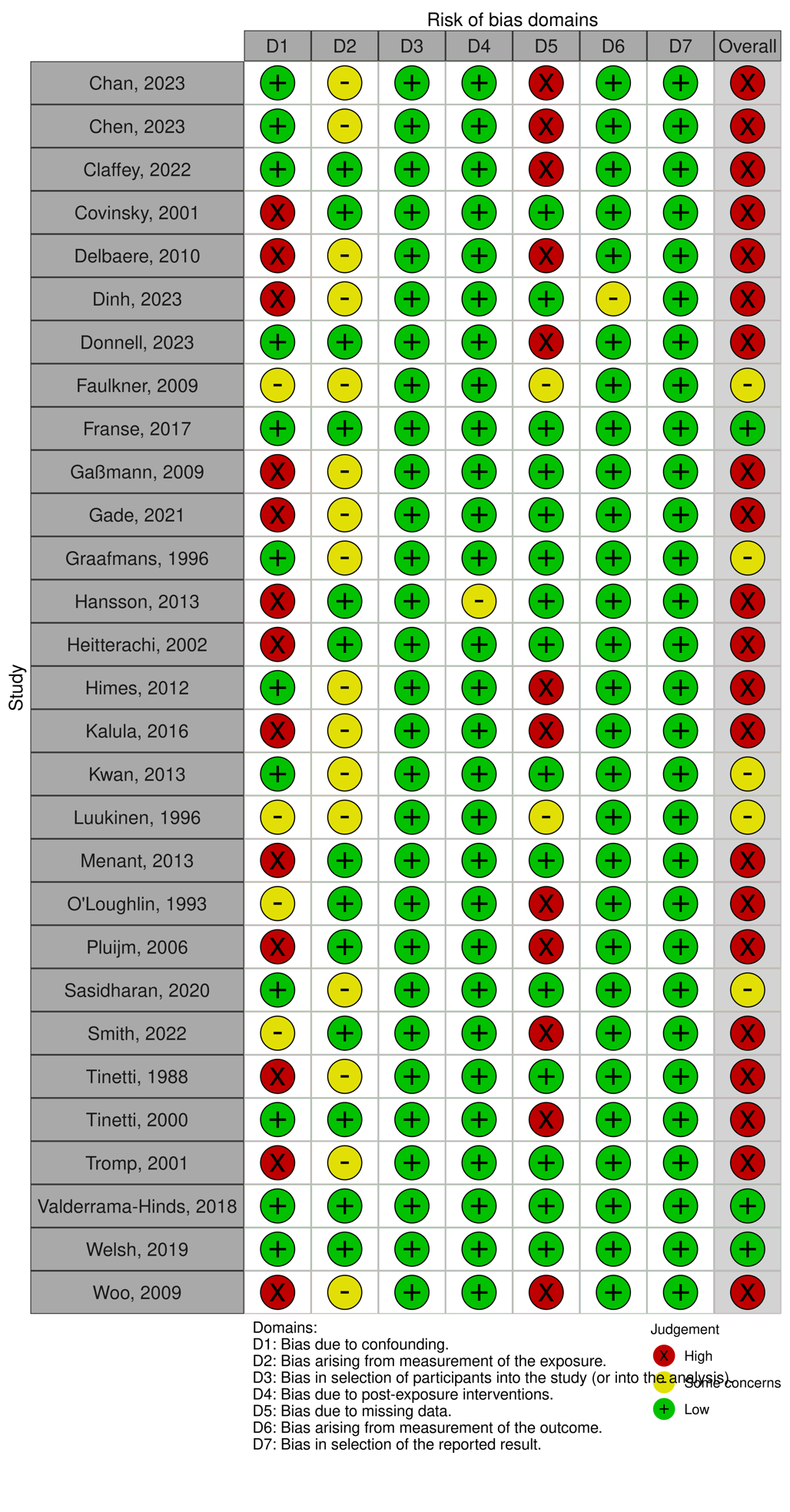


**
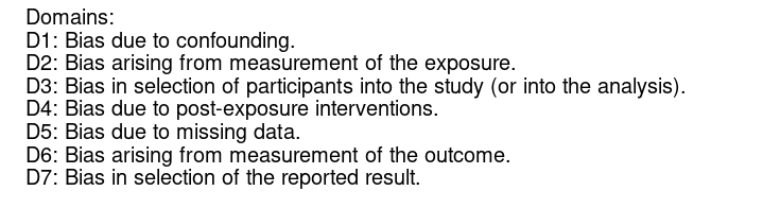

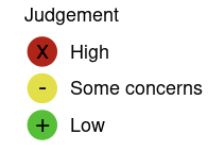
**

**Appendix G. PRISMA guidelines checklist**

| **Section and Topic** | **Item** | **Checklist item** | **Location where item is reported** |
| --- | --- | --- | --- |
| **TITLE** | | |  |
| Title | 1 | Identify the report as a systematic review. | Title page |
| **ABSTRACT** | | |  |
| Abstract | 2 | See the PRISMA 2020 for Abstracts checklist.  **Background:** main objectives  **Methods:** data sources; study eligibility criteria, participants, and interventions; study appraisal; and synthesis methods, such as network meta-analysis.  **Results:** number of studies and participants identified; summary estimates with corresponding confidence/credible intervals; treatment rankings may also be discussed. Authors may choose to summarize pairwise comparisons against a chosen treatment included in their analyses for brevity.  **Discussion/Conclusions:** limitations; conclusions and implications of findings.  **Other:** primary source of funding; systematic review registration number with registry name. | Manuscript Page 1 |
| **INTRODUCTION** | | |  |
| Rationale | 3 | Describe the rationale for the review in the context of existing knowledge. | Page 2 |
| Objectives | 4 | Provide an explicit statement of the objective(s) or question(s) the review addresses. | Page 2, the last paragraph of Introduction |
| **METHODS** | | |  |
| Eligibility criteria | 5 | Specify the inclusion and exclusion criteria for the review and how studies were grouped for the syntheses. | Page 3, ‘Eligibility criteria and study selection’ |
| Information sources | 6 | Specify all databases, registers, websites, organisations, reference lists and other sources searched or consulted to identify studies. Specify the date when each source was last searched or consulted. | Page 2-3, ‘Search strategy and information sources’ |
| Search strategy | 7 | Present the full search strategies for all databases, registers and websites, including any filters and limits used. | Supplementary Appendix A |
| Selection process | 8 | Specify the methods used to decide whether a study met the inclusion criteria of the review, including how many reviewers screened each record and each report retrieved, whether they worked independently, and if applicable, details of automation tools used in the process. | Page 3, ‘Eligibility criteria and study selection’ |
| Data collection process | 9 | Specify the methods used to collect data from reports, including how many reviewers collected data from each report, whether they worked independently, any processes for obtaining or confirming data from study investigators, and if applicable, details of automation tools used in the process. | Page 3, ‘Data extraction’ |
| Data items | 10a | List and define all outcomes for which data were sought. Specify whether all results that were compatible with each outcome domain in each study were sought (e.g. for all measures, time points, analyses), and if not, the methods used to decide which results to collect. | Page 3, ‘Data extraction’ |
|  | 10b | List and define all other variables for which data were sought (e.g. participant and intervention characteristics, funding sources). Describe any assumptions made about any missing or unclear information. | Page 3, ‘Data extraction’ |
| Study risk of bias assessment | 11 | Specify the methods used to assess risk of bias in the included studies, including details of the tool(s) used, how many reviewers assessed each study and whether they worked independently, and if applicable, details of automation tools used in the process. | Page 4, ‘Risk of bias assessment’ |
| Effect measures | 12 | Specify for each outcome the effect measure(s) (e.g. risk ratio, mean difference) used in the synthesis or presentation of results. | Page 4, ‘Statistical analysis’ |
| Synthesis methods | 13a | Describe the processes used to decide which studies were eligible for each synthesis (e.g. tabulating the study intervention characteristics and comparing against the planned groups for each synthesis (item #5)). | Page 4, ‘Statistical analysis’ |
|  | 13b | Describe any methods required to prepare the data for presentation or synthesis, such as handling of missing summary statistics, or data conversions. | Page 4, ‘Statistical analysis’ |
|  | 13c | Describe any methods used to tabulate or visually display results of individual studies and syntheses. | Page 4, ‘Statistical analysis’ |
|  | 13d | Describe any methods used to synthesize results and provide a rationale for the choice(s). If meta-analysis was performed, describe the model(s), method(s) to identify the presence and extent of statistical heterogeneity, and software package(s) used. | Page 4, ‘Statistical analysis’ |
|  | 13e | Describe any methods used to explore possible causes of heterogeneity among study results (e.g. subgroup analysis, meta-regression). | N/A |
|  | 13f | Describe any sensitivity analyses conducted to assess robustness of the synthesized results. | N/A |
| Reporting bias assessment | 14 | Describe any methods used to assess risk of bias due to missing results in a synthesis (arising from reporting biases). | Page 4, ‘Statistical analysis’ |
| Certainty assessment | 15 | Describe any methods used to assess certainty (or confidence) in the body of evidence for an outcome. | Page 4, ‘Statistical analysis’ |
| **RESULTS** | | |  |
| Study selection | 16a | Describe the results of the search and selection process, from the number of records identified in the search to the number of studies included in the review, ideally using a flow diagram. | Page 5, 1^st^ paragraph of Results; and Figure 1 |
|  | 16b | Cite studies that might appear to meet the inclusion criteria, but which were excluded, and explain why they were excluded. | Page 5, 1^st^ paragraph of Results |
| Study characteristics | 17 | Cite each included study and present its characteristics. | Pages 5, second and third paragraphs of Results |
| Risk of bias in studies | 18 | Present assessments of risk of bias for each included study. | Page 7, ‘Quality of included studies’ |
| Results of individual studies | 19 | For all outcomes, present, for each study: (a) summary statistics for each group (where appropriate) and (b) an effect estimate and its precision (e.g. confidence/credible interval), ideally using structured tables or plots. | Pages 5-7 and Tables 1-2 and Figures 2-4 |
| Results of syntheses | 20a | For each synthesis, briefly summarise the characteristics and risk of bias among contributing studies. | Pages 5-7, ‘Main findings’ |
|  | 20b | Present results of all statistical syntheses conducted. If meta-analysis was done, present for each the summary estimate and its precision (e.g. confidence/credible interval) and measures of statistical heterogeneity. If comparing groups, describe the direction of the effect. | Page 6, ‘Main findings’ |
|  | 20c | Present results of all investigations of possible causes of heterogeneity among study results. | N/A |
|  | 20d | Present results of all sensitivity analyses conducted to assess the robustness of the synthesized results. | N/A |
| Reporting biases | 21 | Present assessments of risk of bias due to missing results (arising from reporting biases) for each synthesis assessed. | Supplementary Appendix F |
| Certainty of evidence | 22 | Present assessments of certainty (or confidence) in the body of evidence for each outcome assessed. | N/A |
| **DISCUSSION** | | |  |
| Discussion | 23a | Provide a general interpretation of the results in the context of other evidence. | Page 8, Discussion |
|  | 23b | Discuss any limitations of the evidence included in the review. | Pages 8, the 6^th^ paragraph of Discussion |
|  | 23c | Discuss any limitations of the review processes used. | Pages 8, the 6^th^ paragraph of Discussion |
|  | 23d | Discuss implications of the results for practice, policy, and future research. | last paragraph of Discussion |
| **OTHER INFORMATION** | | |  |
| Registration and protocol | 24a | Provide registration information for the review, including register name and registration number, or state that the review was not registered. | Page 2, ‘Protocol and registration’ of Method |
|  | 24b | Indicate where the review protocol can be accessed, or state that a protocol was not prepared. | Page 2, ‘Protocol and registration’ of Method |
|  | 24c | Describe and explain any amendments to information provided at registration or in the protocol. | N/A |
| Support | 25 | Describe sources of financial or non-financial support for the review, and the role of the funders or sponsors in the review. | Title page |
| Competing interests | 26 | Declare any competing interests of review authors. | Title page |
| Availability of data, code and other materials | 27 | Report which of the following are publicly available and where they can be found: template data collection forms; data extracted from included studies; data used for all analyses; analytic code; any other materials used in the review. | N/A |

*From:*  Page MJ, McKenzie JE, Bossuyt PM, Boutron I, Hoffmann TC, Mulrow CD, et al. The PRISMA 2020 statement: an updated guideline for reporting systematic reviews. BMJ 2021;372:n71. doi: 10.1136/bmj.n71

For more information, visit: <http://www.prisma-statement.org/>
